# Antimetastatic effects of MRTX1133 KRAS G12D specific inhibitor in a liver metastatic model of pancreatic ductal adenocarcinoma

**DOI:** 10.1038/s41598-025-34204-y

**Published:** 2026-01-07

**Authors:** Krisztina Andrea Szigeti, Marcell Baranyi, Sára Surguta, Balázs Hegedűs, Violetta Piurkó, József Tóvári, József Tímár

**Affiliations:** 1 Department of Pathology, Forensic and Insurance Medicine, Semmelweis University, Budapest, 1091 Hungary; 2 KINETO Lab Ltd, Budapest, 1037 Hungary; 3 Department of Experimental Pharmacology and the National Tumor Biology Laboratory, National Institute of Oncology, Budapest, 1122 Hungary; 4 School of Ph.D. Studies, Semmelweis University, Budapest, 1085 Hungary; 5 Department of Thoracic Surgery, University Medicine Essen - Ruhrlandklinik, University Duisburg-Essen, 45239 Essen, Germany

**Keywords:** KRAS, MRTX1133, Metastasis, Pancreatic cancer, Epithelial-mesenchymal transition, Cancer therapy, Metastasis, Oncogenes

## Abstract

MRTX1133 is a first-in-class KRAS G12D inhibitor being in phase I/II clinical trials. KRAS is the most frequently mutated oncogene in pancreatic ductal adenocarcinoma (PDAC), with a predominance of the G12D variant. PDAC is a highly lethal cancer type with an extremely low 5-year survival rate. Although PDAC metastasis most frequently targets the liver, the influence of MRTX1133 treatment on hepatic metastases has not yet been investigated thoroughly. Thus, we aimed to analyze the effect of MRTX1133 treatment on local tumor growth and liver metastasis development. Cell proliferation and migration were investigated in vitro using clonogenic, scratch, Boyden chamber, and immunoblot assay in three KRAS G12D mutated PDAC cell lines (ASPC1, SW1990, PANC1). Moreover, PANC1 was examined in vivo in a spleen-to-liver metastatic xenograft model using NXG female mice. The MRTX1133 treatment decreased clonogenic proliferation and migratory activity; furthermore, it inhibited both local tumor growth in the spleen and liver colonization. MRTX1133 induced alterations associated with mesenchymal-to-epithelial transition; furthermore, lower levels of activated Erk, altered FAK expression, and activation were observed. In addition to the antiproliferative effects of MRTX1133, our in vitro and in vivo results indicate the importance of MRTX1133 as a potential antimetastatic drug in PDAC therapy.

**Supplementary Information:**

The online version contains supplementary material available at 10.1038/s41598-025-34204-y.

## Introduction

Pancreatic ductal adenocarcinoma (PDAC) is one of the most aggressive cancers, ranking as the sixth leading cause of cancer-related deaths in industrialized countries^1^. About 80% of the patients are diagnosed with advanced stages since most cases show no definite symptoms until reaching the state of locally dispersed or metastatic tumors, from which the liver is the most commonly affected organ for PDAC progression^2–6^. Furthermore, most of the resectable cases eventually endure distant recurrence^7^, while potential treatments – especially targeted therapies – are currently limited^8,9^. These factors altogether lead to a dismal prognosis of PDAC cases with a 5-year survival rate of only about 10%^2^. Hence, in order to significantly improve survival rates, the development of new therapeutic strategies is crucial.

In the case of non-resectable tumors, the standard chemotherapeutic treatment involves FOLFIRINOX and gemcitabine as a monotherapy or in combination with nab-paclitaxel, from which FOLFIRINOX shows a better survival rate compared to gemcitabine; however, it is associated with higher levels of toxicity^10–12^. Besides, NALIRIFOX is also available as first-line treatment, showing better overall survival compared to gemcitabine plus nab-paclitaxel^13^.

Various therapies targeting BRCA1/2, HER2, EGFR, and IDH1 are also explored in PDAC treatment, yet their efficacies are limited (reviewed in^14^ and^15^). Moreover, PDAC is prone to rapidly develop therapy resistance ^16^.

The most common genetic mutations in PDAC formation affect *KRAS, CDKN2A, TP53,* and *SMAD4* genes^15,17^. As *KRAS* mutation is one of the earliest crucial oncogenic alterations during PDAC development, it can serve as a potential target^10,18,19^. For long, mutated KRAS variants were labeled as undruggable until the characterization of a new potential drug-binding site (switch II pocket) of KRAS proteins in 2013^20^. This essential milestone was shortly followed by the discovery of effective inhibitors against KRAS G12C mutated protein^21,22^, which—as a proof of concept—has opened a new era in the research of targeted cancer therapy^21–27^. Among these new compounds, MRTX1133 is a first-in-class, promising anticancer drug specific to KRAS G12D^28,29^, the most frequent mutant KRAS variant in PDAC with about 50% prevalence^30–32^. This small-molecule inhibitor interacts in a non-covalent manner with the switch II pocket of KRAS-G12D proteins with a picomolar binding affinity and high selectivity over wild-type KRAS^28,29^. While several other KRAS G12D-targeting candidates—like INCB 161,734, LY3962673, RMC-9805, and HRS-4642—are in clinical trials, and the latter two drugs have already shown promising anti-tumoral activity, MRTX1133 is the most extensively studied in preclinical experiments (reviewed in^33–35^).

Previous studies demonstrated that MRTX1133 can interfere with KRAS-G12D function^28,29,36–38^. Inhibition of KRAS-mediated signaling pathways and significant effects on cell viability were observed using MRTX1133 in nanomolar concentrations investigating different cell lines^28,29^. Moreover, evaluation of in vivo experiments showed dose-dependent antitumor activity in cell-line-derived and patient-derived xenograft models, where tumors were subcutaneously implanted^28,29^. Experiments using MRTX1133 monotherapy for immunocompetent mice models injected with syngeneic PDAC cells in an orthotopic manner also revealed high treatment efficacy^37^. Furthermore, MRTX1133 was previously shown to modulate the tumor microenvironment (TME) by reprogramming CD8+ cells, promoting enhanced immune responses^38^, or altering fibroblast activation, ECM composition, or the presence of macrophages^37^. Notably, the latter study also showed that the presence of T-cells was necessary for maintaining anti-tumoral responses, emphasizing the impact of MRTX1133 on immune surveillance^37^. This inhibitor was already studied not just as a monotherapy but in combination with drugs applied in clinical treatment, like HER family inhibitors^28,36^, PI3Kα inhibitor^28^, immune checkpoint inhibitors^38^, or farnesyl-transferase inhibitors^39^. However, even though promising results were published about the tumor-reducing effect of MRTX1133 in syngeneic mouse models^37^, also in cases of locally advanced stages^38^, no study has been published investigating the anti-metastatic capacity of MRTX1133 mirroring the organotropism of PDAC, more specifically, liver metastasis. Previous studies focused on lung metastatic models, showing varying potency of MRTX1133 on the inhibition of PDAC lung colonization^37,40^.

Thus, we aimed to analyze the impact of MRTX1133 treatment on cell migration in vitro and metastasis development in a liver metastatic mouse model in vivo^41^.

## Results

### The influence of MRTX1133 on cell proliferation

**Fig. 1:**
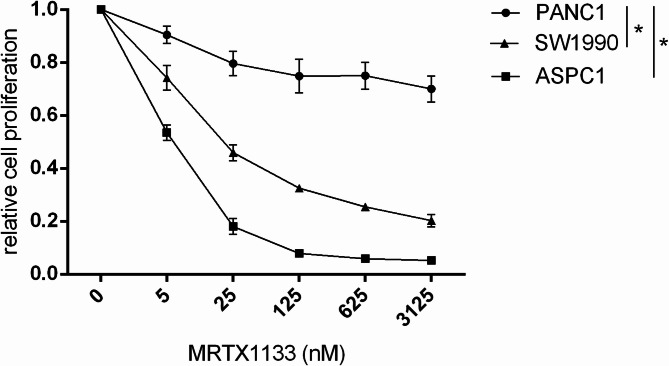
Cell proliferation of ASPC1, SW1990, and PANC1 cell lines in response to MRTX1133 treatment for two doublings. MRTX1133 concentrations of 0, 5, 25, 125, 625, 3125 nM were applied. MRTX1133 sensitivity was significantly higher in ASPC1 and SW1990 cells compared to PANC1 samples (*p ≤ 0.05). Doubling time of PANC1 was 2 days, and 4 days for SW1990 and ASPC1. Data represent the mean ± SEM of three replicates.

First, doubling time was defined for all the cell lines. PANC1 cell population doubled in 2 days, SW1990 and ASPC1 cells in 4 days. The impact of MRTX1133 treatment on cell proliferation was investigated with SRB assay for two doublings (PANC1: 4 days, SW1990, ASPC1: 8 days). Significantly higher sensitivity was detected in the case of ASPC1 and SW1990 in comparison with PANC1 cells (*p* ≤ 0.05) (Fig. 1). IC50 values could be calculated in the case of ASPC1 (5,6 nM) and SW1990 cells (31,3 nM) as the viability curve exhibited a plateau before reaching 50% inhibition in the case of PANC1.

### The effect of MRTX1133 treatment on clonogenic potential

The long-term drug effect on clonogenic growth of ASPC1, SW1990, and PANC1 cell lines was investigated by applying MRTX1133 treatment for 10 days. As IC50 values could not be calculated for all the cell lines due to a plateau in the viability curves even in micromolar range in PANC1, 25 nM—the second lowest concentration from the short-term viability assays—was chosen for these long-term experiments.

**Fig. 2:**
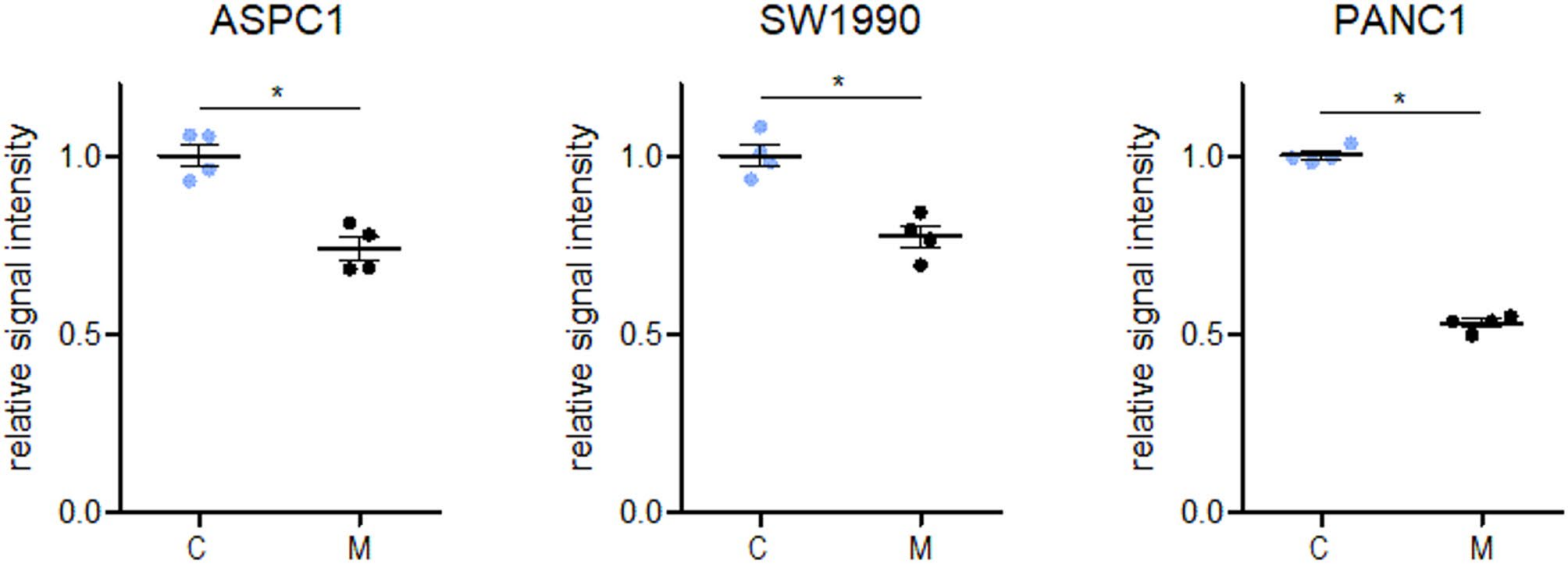
Alteration in clonogenic growth of ASPC1, SW1990, and PANC1 cell lines in response to 25 nM MRTX1133 treatment for 10 days. Significantly decreased proliferation was detected in all three cell lines (*p ≤ 0.05) following MRTX1133 application. Data represent the mean ± SEM of four replicates. C: control samples, M: MRTX1133 treated specimens.

Significant inhibition of clonogenic growth (*p ≤ 0.05) was observed in all three cell lines upon MRTX1133 treatment (ASPC1: 0.74 ± 0.07, SW1990: 0.77 ± 0.03, PANC1: 0.53 ± 0.02) compared to the control specimens (ASPC1: 1.0 ± 0.07, SW1990: 1.0 ± 0.03, PANC1: 1.0 ± 0.02) (Fig. 2). Raw OD values of the control wells showing cell line-specific clonogenic properties are presented in Supplementary Fig. 1.

### The influence of MRTX1133 treatment on cell migration

**Fig. 3:**
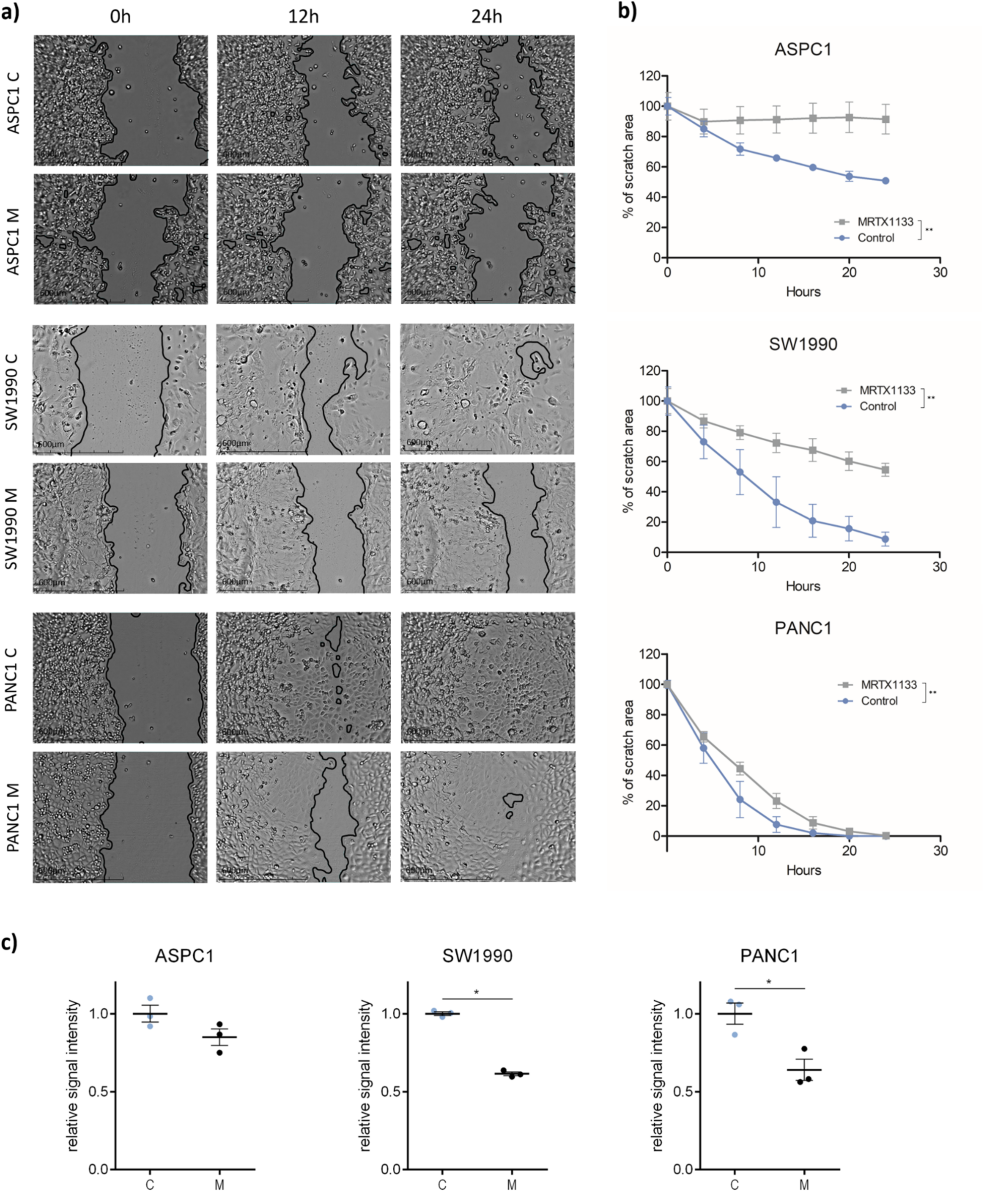
Changes in migratory capacity of ASPC1, SW1990, and PANC1 cell lines prompted by MRTX1133 treatment. (**A**) Representative images of scratch assay experiments taken at 0 h, 12 h, and 24 h during the experiment. Following a one-day-long pre-treatment with 100 nM MRTX1133**,** wound closure was investigated for 24 h under the same treatment concentration while images were captured every hour. (**B**) Normalized data of scratch assay showing significantly slower wound closure in all the investigated cell lines upon treatment (**p ≤ 0.01). (**C**) Results of Boyden chamber experiments. Following a one-day-long pre-treatment with 100 nM MRTX1133**,** migration in Boyden chamber for 4 h under the same treatment concentration was measured. MRTX1133 significantly inhibited migratory activity of SW1990 and PANC1 (* ≤ *p0.05*). C: control samples, M: MRTX1133 treated specimens.

The effect of MRTX1133 treatment on in vitro cell migration was examined by using scratch assays. The treatment induced significantly lower (**p ≤ 0.01) wound closure capacity in all the investigated cell lines compared to untreated samples (Fig. 3A, B). T_1/2_ wound closure rates were 24.21, 7.84, and 4.89 h for control ASPC1, SW1990, and PANC1 samples, which values were extended to 30.75 and 6.64 following MRTX1133 treatment of SW1990 and PANC1, respectively, while in the case of treated ASPC1, wound closure was found to be completely halted. Wound closure of ASPC1 and SW1990 was profoundly inhibited, while in the case of PANC1, MRTX1133 showed a modest, albeit significant difference in response to drug application.

In order to complement the scratch assay results, we conducted Boyden assay to further investigate the anti-migratory effects of MRTX1133 treatment (Fig. 3C). MRTX1133 treatment significantly inhibited 3D migration in Boyden assay in the case of SW1990 and PANC1 cells, while in ASPC1, only numerical decrease can be observed. Notably, ASPC1 exhibited only minimal migratory activity in both scratch and Boyden assays. Representative images of Boyden chamber assay are shown in Supplementary Fig. 2.

### *The impact of MRTX1133 treatment on protein expression of cell proliferation, epithelial-mesenchymal transition*,* and cell migration markers*

**Fig. 4:**
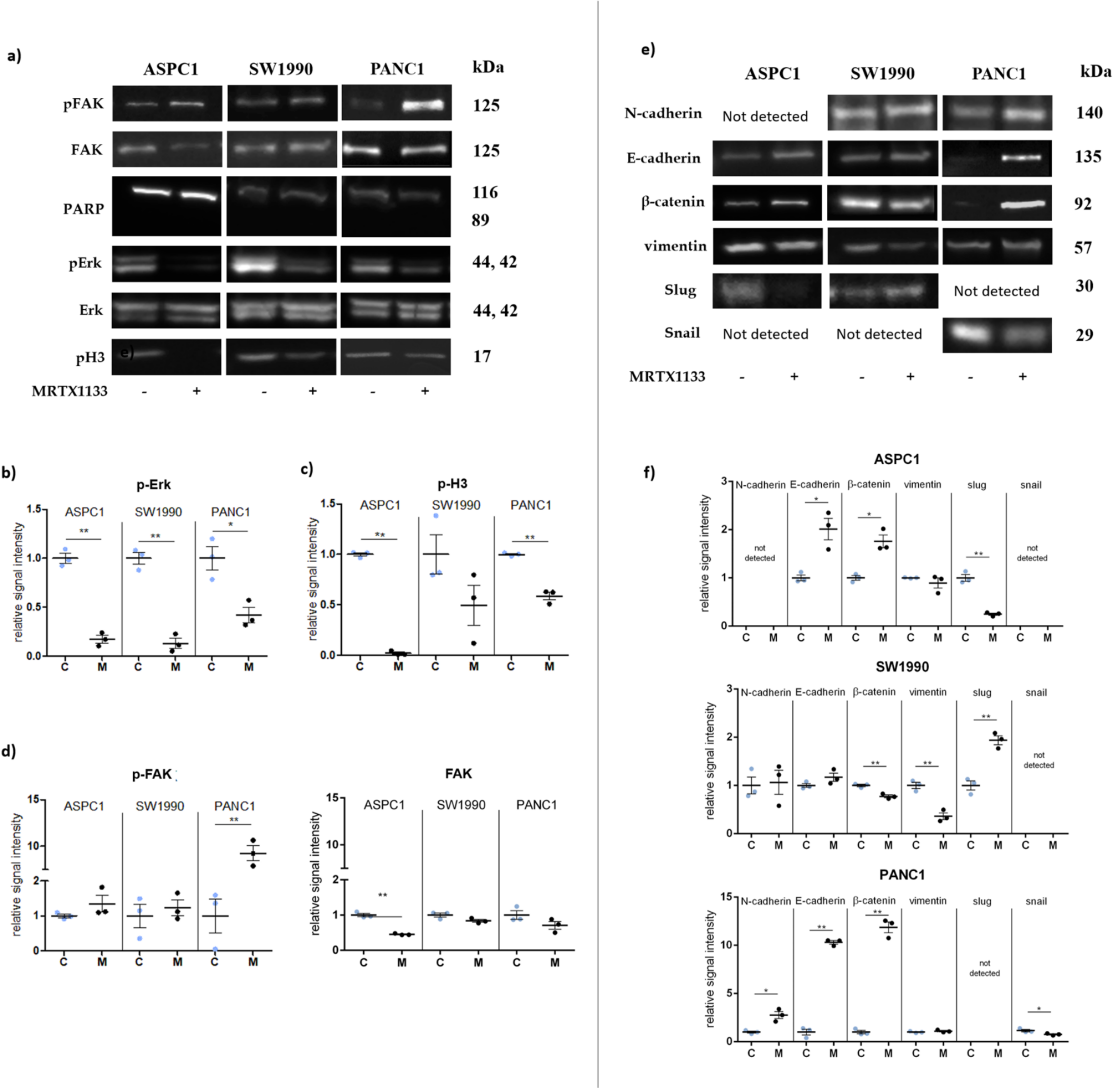
Immunoblot analyses of changes in the expression of proteins connected with proliferation, epithelial-mesenchymal transition, and migration upon 100 nM MRTX1133 treatment for 48 h. The figure shows results from ASPC1, SW1990, and PANC1 pancreatic adenocarcinoma cell lines. (**A**) and (**E**) Representative images of the immunoblot results are adjusted for better visualization. (**B**–**D**) and (**F**) Graphs indicate normalized data of protein expression differences. Significant changes are depicted with asterisks: **p ≤ 0.01, *p ≤ 0.05. Data represent the mean ± SEM of three replicates. C: control samples, Erk: extracellular signal-regulated kinase 1/2, FAK: total focal adhesion kinase, M: MRTX1133 treated specimens, p-Erk: phospho-extracellular signal-regulated kinase 1/2, p-FAK: activated focal adhesion kinase, p-H3: phospho-histone H3, PARP: poly-ADP Ribose Polymerase.

The effect of MRTX1133 treatment on Ras-signaling was investigated by the analysis of p-Erk expression using western blot. Significantly lower p-Erk level was detected in ASPC1, SW1990, and PANC1 cells treated with MRTX1133 compared to the control specimens (**p ≤ 0.01, *p ≤ 0.05; Fig. 4B).

To explore the alteration in cell proliferation in response to MRTX1133 treatment, a proliferation marker, p-H3 expression was analyzed by western blot experiments. The expression of p-H3 showed a significant decline in the MRTX1133-treated ASPC1 and PANC1 samples in comparison with the untreated specimens (**p ≤ 0.01; Fig. 4C).

PARP immunoblot was also performed to detect possible pro-apoptotic effects of MRTX1133, however, no observable amount of cleaved PARP was detected following MRTX1133 treatment (Fig. 4A).

Proteins connected to the process of metastasis formation were also investigated using immunoblot analyses. The amount of activated and total FAK was evaluated to explore the impact of MRTX1133 treatment on cell migration. In response to the application of MRTX1133, significantly (**p ≤ 0.01) upregulated p-FAK level was observed in the PANC1 samples. Furthermore, no alteration in p-FAK but significantly reduced FAK expression (**p ≤ 0.01) was detected in the case of ASPC1 cells following the treatment (Fig. 4D).

Moreover, N-cadherin, E-cadherin, Snail, Slug, and vimentin, along with β-catenin were measured to observe changes in protein expression related to EMT. Notably, baseline expressions of these markers were substantially different between cell lines (Supplementary Fig. 11). Among these markers, E-cadherin showed the greatest variance, being strongly expressed in SW1990, moderately expressed in ASPC1, and weakly expressed in PANC1. Expression of β-catenin was proportional to E-cadherin expression in all cell lines. Baseline expression of N-cadherin was weak in PANC1 cell line alongside with strong vimentin and moderate Snail expression. Slug expression was not detected in PANC1. ASPC1 did not express N-cadherin, while expression of vimentin was similar to PANC1. ASPC1 only expressed Slug, while it showed no detectable Snail expression. SW1990 had weak N-cadherin and vimentin signals. Snail was not detected in this cell line, while expression of Slug was similar to ASPC1.

E-cadherin showed drastically higher expression levels in treated PANC1 cells (~ tenfold), and a more modest elevation in ASPC1 cells in comparison with the controls (**p ≤ 0.01, *p ≤ 0.05) (Fig. 4F). Interestingly, while N-cadherin was elevated in PANC1 cells, it showed no alteration in SW1990 upon treatment. N-cadherin could not be detected in ASPC1 cells even after MRTX1133 treatment (Fig. 4F). Significant elevation of β-catenin expression was noticed in treated ASPC1 and PANC1 cells, while significantly decreased protein level was observed in treated SW1990 compared to control specimens (**p ≤ 0.01, *p ≤ 0.05; Fig. 4F). Notably, the increase in β-catenin expression in PANC1 cells was comparable to that observed in the case of E-cadherin (~ tenfold). Vimentin was significantly downregulated in SW1990 in MRTX1133 treated samples compared to control specimens (**p ≤ 0.01), while no statistically significant alteration was observed in ASPC1 and PANC1 cell lines (Fig. 4F). Snail expression could be observed only in PANC1 cells with a significantly decreased level upon treatment, while Slug showed a significant reduction in ASPC1, significant elevation in SW1990 cells, and it could not be detected in PANC1 (Fig. 4F).

Representative images of the immunoblot assay are shown in Fig. 4A and E; unmodified images are depicted in Supplementary Materials.

### *The effect of MRTX1133 on splenic tumor growth and liver metastasis formation *in vivo

Following the in vitro experiments, the effect of 10 mg/kg MRTX1133 treatment on splenic tumor growth and colonization capacity was investigated in xenograft models as well, where PANC1 cells were injected into the spleen. We observed no sign of toxicity based on changes in mice weight and histophatological analysis of the liver tissue for structural integrity following termination (Supplementary Figs. 3).

**Fig. 5:**
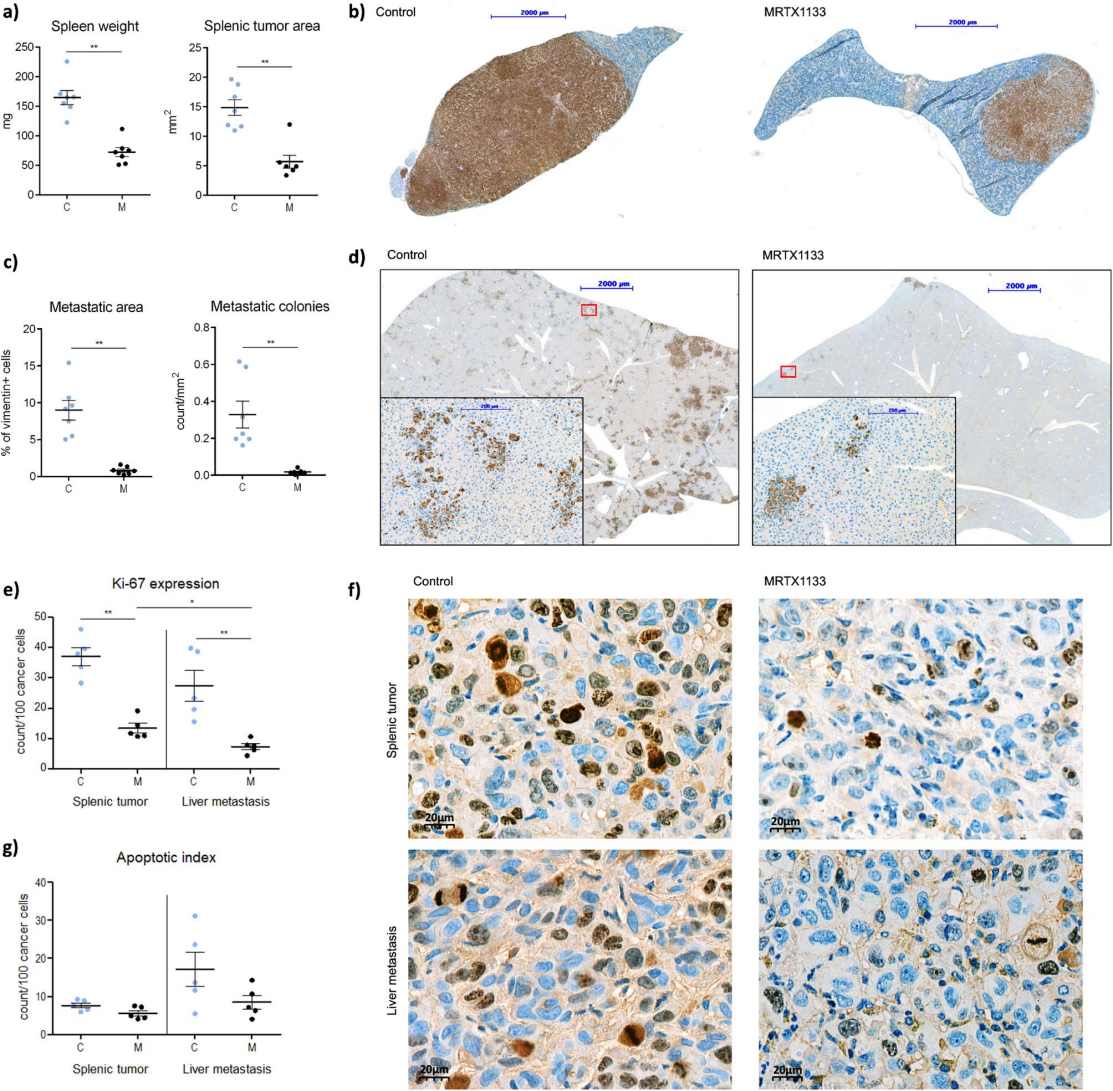
The influence of MRTX1133 on PANC1 splenic tumor growth and metastatic capacity. Treatments started 7 days after tumor cell injection with 10 mg/kg MRTX1133 dose, 5 times per week for 22 days. The mice in the control group were treated with the solvent. Termination of the experiment was 29 days after cell inoculation. (**A**) Significantly lower spleen weight (**p ≤ 0.01, left) and significantly smaller splenic tumors (*p ≤ 0.05, right) were found in the MRTX1133 treated group compared to untreated animals. (**B**) Vimentin staining of representative spleen sections with splenic tumor areas of control (left) and MRTX1133 treated animals (right). (**C**) Significantly smaller metastatic lesions (**p ≤ 0.01, left) and a significantly reduced number of metastatic colonies (**p ≤ 0.01, right) were detected in the livers of MRTX1133 treated animals compared to the controls. (**D**) Vimentin staining of representative metastatic liver sections of control (left) and MRTX1133 treated animals (right). (**E**) A significantly lower Ki67 index was detected in the splenic tumor of treated animals compared to the control ones, both in splenic tumor and in liver metastasis. (**p ≤ 0.01,*p ≤ 0.05). (**F**) Representative images of Ki67 expression. (**G**) Investigation of apoptotic bodies revealed no significant induction of apoptosis following MRTX1133 treatment. Magnified areas depicted on the bottom are indicated by red boxes. Data represent the mean ± SEM of seven animals. C: control samples, M: MRTX1133 treated specimens.

Significantly lower spleen weights (**p ≤ 0.01) (Fig. 5A, left) and significantly smaller splenic tumors were detected based on vimentin immunohistochemistry staining (*p ≤ 0.05) (Fig. 5A, right; Supplementary Fig. 4) in the case of treated mice compared to control animals. Representative images of splenic tumors are shown in Fig. 5B.

Measurement of liver weights did not reveal any significant differences (Supplementary Fig. 3b). Liver metastases have also been evaluated with various morphometric techniques. Uniform expression of vimentin was found in PANC1 cells, making it an excellent tumor-specific marker in mouse liver specimens. Notably, no signal was detected for E-cadherin in PANC1 tumors, either in control or in MRTX1133 treated groups. Following investigation of liver specimens, we observed a significantly lower percentage of tumor cells in response to MRTX1133 treatment based on vimentin-positive cells (**p ≤ 0.01; Fig. 5C, left). Moreover, the number of metastatic colonies also decreased significantly in the MRTX1133 treated animals compared to the control ones (**p ≤ 0.01; Fig. 5C, right). Besides, vimentin results were confirmed by analyses on hematoxylin–eosin-stained slides with manual training for metastatic cells in Qupath software (Supplementary Fig. 5). Representative images of liver metastases are shown in Fig. 5D.

Further analyses were performed to investigate the effects of MRTX1133 treatment on cellular processes as well as the tumor microenvironment.

Investigation of proliferation using Ki67 immunohistochemical staining revealed that in the control mice group the spleen primary tumors have a relatively high ratio of cells that entered cell cycle, while this ratio was lower in the liver metastases (Fig. 5E, F, Supplementary Fig. 6). MRTX1133 significantly reduced Ki67 positive tumor cell ratio both in the splenic and the metastatic tumors, compared to the control ones. Furthermore, in the MRTX1133 treated mice group, metastatic lesions exhibited significantly lower Ki67 score compared to splenic tumors (Fig. 5E, F). Effects on proliferation were further confirmed by determination of mitotic bodies, showing significantly lower mitosis in MRTX1133 treated mice both in the splenic tumors and in the metastatic lesions (Supplementary Fig. 6).

Investigation of apoptotic bodies revealed no significant induction of apoptosis following MRTX1133 treatment (Fig. 5G).

ECM composition and fibroblast presence were studied by Mallory’s trichrome and Picrosirius Red staining. Primary spleen tumors contained a low amount of intratumoral ECM as detected by Mallory stain (blue stain), and it was almost completely missing from liver metastasis (Supplementary Fig. 7). MRTX1133 treatment had no effect on ECM content either in primary tumor or in liver metastasis (Supplementary Fig. 7). However, primary spleen tumors contained a rich intratumoral collagen matrix detected by Picrosirius Red staining which was also present in liver metastases, although no alterations could be detected following MRTX1133 treatment (Supplementary Fig. 8).

NXG immunodeficient mice lack functional T, B and NK cells, thus investigation of the changes in the adaptive immune system was not feasible. Accordingly, intratumoral mononuclear cells investigated in this model are primarily macrophages and/or leukocytes. In untreated PANC1 spleen primary tumors and in their liver metastases the tumor infiltrating macrophage (TIM) ratio was relatively low compared to tumor cells (Supplementary Fig. 9). MRTX1133 treatment induced a significant increase in TIM-tumor cell ratio in the primary and even more profoundly, in the metastatic tumor tissues (Supplementary Fig. 9).

Furthermore, p-Erk level of splenic tumors in response to MRTX1133 treatment decreased (Supplementary Fig. 10).

## Discussion

Mutations of KRAS can be found in about 90% of the PDAC cases^30,32^. The discovery of an efficient inhibitor specific to KRAS-G12C was a breakthrough in targeted cancer therapy^20–22,24,26,28,29^, in part paving the way to the development of MRTX1133^28,29^. This small-molecule drug is a potent, first-in-class inhibitor against the KRAS G12D variant^28,29^, which can be found in about 50% of PDAC cases^30–32^. Since the first publication of MRTX1133, several studies have investigated the effect of MRTX1133 treatment on cell proliferation, KRAS-mediated molecular pathways, tumor growth, and resistance mechanisms^28,29,37,38,42^. Moreover, the combination of farnesyl-transferase inhibition with MRTX1133 treatment was analyzed by our group^39^. Despite PDAC being one of the most invasive cancers^2^, the impact of MRTX1133 on distant metastases has been less of a center of the analyses yet^37,38^. Thus, in this study, we aimed to examine the effect of MRTX1133 treatment on local tumor growth and liver metastasis development, investigating three KRAS-G12D mutated cell lines (ASPC1, SW1990, PANC1).

In line with previous findings^28,39^, the cell proliferation of ASPC1 and SW1990 was remarkably affected by MRTX1133 treatment, although significantly lower sensitivity was observed in PANC1 cells compared to ASPC1 and SW1990 samples. The decreased clonogenic growth and reduced p-H3 expression detected in this study support the antiproliferative effects of MRTX1133. Furthermore, in line with previous findings^28,37^, neither in vitro immunoblot analysis nor in vivo counting of apoptotic bodies shows pro-apoptotic effects of MRTX1133, suggesting that it mainly acts through inhibition of proliferation.

Interestingly, PANC1 cells showed resistant phenotype to MRTX1133 in the short-term viability assay, but they showed the strongest response to the treatment in the long-term clonogenic assay compared to the other two cell lines. Although these results may appear paradoxical, the difference can be explained by the distinct biological processes assessed by the two assays. In the case of clonogenic assay, clonogenic potential under low cell-density conditions is investigated using a low treatment concentration for a longer period of time. In our experience, PANC1 cells have far the strongest clonogenic potential from single cell clones compared to SW1990 and ASPC1, which seem to require higher cellular density and probably cell–cell interactions for maintaining proliferation (Supplementary Fig. 1). Anti-proliferative properties of MRTX1133 thus were more obvious with the highly clonogenic PANC1 cells compared to ASPC1 and SW1990 with significantly less proliferation under low cell density conditions.

Previous studies investigated the potential influence of whether the zygosity of the KRAS mutations can affect sensitivity towards KRAS targeting. Notably, PANC1 harbors heterozygous, while ASPC1 and SW1990 harbor homozygous KRAS G12D mutations. Our context-dependent results (e.g. distinct outcomes for PANC1 in short-term viability and clonogenic experiments, as well as in vivo results) outline prior findings with no clear association between zygosity of KRAS mutations and sensitivity to KRAS-specific inhibitors^43,44^.

Metastasis is a multistep process in which primary tumor cells undergo EMT and gain abilities to invade the surrounding tissue and, through the vascular or lymphatic system, disseminate to distant organs, where eventually colonization can occur^45^. To investigate the role of MRTX1133 in metastases development in PDAC, in vitro and in vivo experiments were conducted. Investigation of cellular motility by scratch assay revealed significantly reduced pace of wound closure in all the investigated PDAC cell lines. In order to minimize the effects of proliferation on the results, we created narrow wounds that closed within 24 h. Furthermore, to complement scratch assay results, we also performed a three-dimensional Boyden chamber assay experiment. Results of Boyden assays confirmed scratch assay results, showing significantly reduced migratory activity following MRTX1133 treatment of SW1990 and PANC1 cells. A statistically not significant decrease was detected in ASPC1 motility; however, ASPC1 showed only minimal motility in both migratory assays.

As migration experiments showed that MRTX1133 can exhibit significant anti-migratory effects besides its known anti-proliferative effects, we sought to investigate its anti-metastatic potential using a relevant in vivo model.

To date, the effect of MRTX1133 on metastatic spread has been only analyzed in lung colonization models, showing variable results. Although Kemp and colleagues demonstrated that MRTX1133 treatment significantly reduced lung colonization following tail vein injection of PDAC cells^37^, in the findings of Dilly et al., MRTX1133 as adjuvant monotherapy could not achieve statistically significant anti-metastatic effects using a spontaneous lung-metastasis model, only if given in a neoadjuvant setting or in combination with chemotherapy^40^.

Liver is the most common metastatic site for PDAC^4–6^ counting up to 60–80% of cases, followed by lung metastases (5–20%)^46^. In this study, using a liver metastatic mouse model with splenic injection of PANC1 cells, we sought to investigate the impact of MRTX1133 on PDAC metastasis formation in vivo in a metastatic model that mirrors organotropism of PDAC^41^. Although several studies performed splenectomy shortly after tumor cell injection, we opted to keep the spleens with primary tumors, as PDAC is often irresectable, and metastatic cases are mostly treated with systemic therapy only.

Significantly smaller splenic tumors were observed in the case of MRTX1133 treated mice in connection with the significantly lower mitotic index observed both on the splenic and metastatic sites. Furthermore, analysis of Ki67—a marker showing cells that entered cell cycle—showed that MRTX1133 treatment significantly reduced the number of Ki67-positive cells both in the splenic tumors and in the metastatic lesions. Interestingly, the percentage of Ki67 positive cells was in general lower in the metastatic tumors both in the control and the MRTX1133 treated animals, although the difference was only significant in the latter group. These results support the antiproliferative effects of MRTX1133 detected in vitro as well.

Moreover, we showed that MRTX1133 treatment strikingly inhibited both the number of metastatic colonies in the liver and the overall ratio of metastatic area to liver area compared with untreated animals. Although decreased proliferation and reduced primary tumors could serve as a reason for smaller metastatic areas observed in response to drug application, it should be noted that MRTX1133 treatment reduced the number of metastatic colonies at a remarkably higher extent, than the ratio of the volume of control versus MRTX1133 treated splenic tumors (ratio of number of liver metastasis 15:1; ratio of splenic tumor volume 3.5:1, Supplementary Fig. 4). These results suggest that MRTX1133 has a specific anti-metastatic effect besides its antiproliferative one in KRAS G12D mutant PDAC cells, strengthening the role of KRAS G12D targeting in the prevention of metastasis formation. In addition, our results support the concept of several studies about KRAS mutation bearing a predictive potential for metastasis development in CRC^47–53^. Accordingly, based on our results, besides its driver oncogene role^15,17,54^, KRAS-G12D may also be a metastasis-promoting oncogene in PDAC progression.

The identification of genes and molecular mechanisms associated with PDAC liver metastasis formation possesses great interest and serves as a goal for several studies in the recent literature^54–61^. For instance, efferocytosis can contribute to PDAC liver metastasis development through macrophage reprogramming^61^; moreover, the role of genes like DYRK2, ITGA3, PAK2, ITGB1, SPARC, TPM1, and mutant KRAS has been published^56–59,62,63^.

Prior studies showed that MRTX1133 may interfere with the tumor microenvironment including cancer associated fibroblasts and CD8+ cells in immunocompetent models^38^. We also sought to investigate if MRTX1133 affects TME in our immunodeficient NXG model. A remarkable amount of fibrillar collagen was found in both splenic tumors and metastatic lesions, although the latter showed lower levels. However, in contrast with previously published results^37,38^, we did not find significant alterations in ECM composition or the amount of fibrillar collagen associated with fibroblast activity following MRTX1133 treatment.

Although NXG mice lack mature T and B cells as well as NK cells, we observed notable presence of mononuclear cells in the PANC1 tumors. Evaluation of the TIM to tumor cell ratio revealed a significantly higher amount of mononuclear cells in the tumor of MRTX1133 treated mice compared to control mice. Moreover, TIM score was significantly higher in the metastatic lesions compared to the splenic tumors in MRTX1133 treated groups. The presence of mononuclear cells likely marks inflammatory responses induced by the treatment.

To further analyze the underlying molecular changes in response to MRTX1133 application in tumor cells, the expression and activation of Erk and FAK were analysed. Besides, changes in the expression of several EMT markers, specifically N- and E-cadherin, β-catenin, vimentin, and transcription factors Snail and Slug were investigated in this study. FAK is an important regulator of cell motility (reviewed in^64^). MRTX1133 treatment interfered with FAK expression and phosphorylation, more specifically, it initiated hyperphosphorylation of FAK at Tyr397 in PANC1 cells. SW1990 showed no significant changes in FAK activation or expression; however, in ASPC1, the level of total FAK was reduced. Changes in FAK activation in PANC1 are similar to our previous findings using KRAS G12C inhibitors, where sotorasib treatment increased Tyr397 phosphorylation of FAK in two out of three KRAS G12C cell lines in a parallel drop of migratory activity^27^. These seemingly paradoxical effects may be explained by the dynamic nature of focal adhesions during cellular migration. Focal adhesions (regulated by FAK) are in a constant assembly-disassembly cycle, making focal contacts at the leading edge of the migrating cells, while conducting disassembly of previously established focal adhesions at the lagging edge of the cells that is necessary for moving forward. Hyperphosphorylation, or even increased phosphorylation of FAK may mark interference with the dynamism of focal adhesions, leading to decreased motility^64–66^.

Reduced p-Erk levels were detected in all the cell lines, which may contribute to the inhibition of cell migration since activated Erk dimerization is proven to have a direct influence on cell motility, as many targets of phosphorylated Erk have key roles in the molecular machinery of cell migration^67–69^.

Investigation of N-cadherin, E-cadherin, β-catenin, vimentin, Snail, and Slug shows that MRTX1133 treatment induced changes in all three PDAC cells connected to mesenchymal-epithelial transition (MET). Higher vimentin level and the loss of E-cadherin expression have been previously associated with increased tumor invasiveness^70–73^. Thus, decreased vimentin level in SW1990 and increased E-cadherin amount in PANC1 and ASPC1 cells after MRTX1333 treatment are consistent with the lower migratory activity observed in this study. Interestingly, the extent of E-cadherin induction appeared inversely related to its basal expression level. PANC1 cells, which expressed low baseline E-cadherin, showed approximately a tenfold increase upon treatment, while ASPC1 cells with moderate expression exhibited a threefold increase. In contrast, SW1990 cells with high basal E-cadherin showed no notable change. Moreover, baseline expression of β-catenin as well as changes following treatment were proportional to E-cadherin levels in all the cell lines. Noteworthy, E-cadherin expression also affects carcinogenesis via its interaction with β-catenin (reviewed in^74^). β-catenin is a component of cell–cell adhesions as part of cadherin/catenin complexes, which connect E-cadherin molecules and the actin filaments of the cytoskeleton^75^. The phosphorylation of β-catenin leads to lower binding affinity to cadherin molecules^76,77^. So, the recovery of cell–cell adhesion (epithelial phenotype) may occur by the increased E-cadherin expression and by the elevated non-phospho-β-catenin level contributing to intact cadherin/catenin complexes. Besides, N-cadherin was also significantly elevated in PANC1 following treatment, although at a much lesser extent compared to E-cadherin. In general, cadherins in PANC1 were elevated from weak baseline expression substantially, suggesting increased cell–cell connections and a shift to a more epithelial expression pattern.

Slug and Snail are transcription factors involved in EMT that can negatively regulate epithelial markers like E-cadherin^78–81^. Interestingly, baseline expressions of Snail and Slug were mutually exclusive in our study, as PANC1 only expressed Snail, while Slug expression could be detected in ASPC1 and SW1990 only. A slight but significant decrease of Snail expression could be observed in PANC1, while Slug decreased significantly in ASPC1 consistent with changes in E-cadherin levels. In SW1990, which showed the highest expression of E-cadherin, significant increase in Slug expression did not lead to decrease of E-cadherin levels.

In summary, MRTX1133-induced MET-related changes were proportional to the baseline expression profile of EMT markers. The most pronounced alterations were observed in PANC1 cells, which exhibit a more mesenchymal expression pattern (low E-cadherin, high vimentin, and moderate Snail expression), followed by ASPC1 cells with an intermediate phenotype (moderate E-cadherin, high vimentin, and moderate Slug expression). In contrast, changes in SW1990 cells, characterized by a more epithelial expression pattern, were limited to decreased vimentin and increased Slug expression, with no detectable change in E-cadherin levels.

In conclusion, our study, utilizing in vitro and in vivo experiments, suggests that MRTX1133 has a specific anti-metastatic potential besides its antiproliferative effect. Mechanistic investigation by immunoblot analysis revealed MET-related changes and interference with FAK signaling as possible mechanisms underlying the observed anti-migratory and anti-metastatic potential of MRTX1133 in a relevant in vivo liver metastasis model. These results also suggest that KRAS G12D oncogenic mutation may have a crucial role not just in PDAC initiation but also in liver metastasis development.

## Methods

### Cell lines and culture conditions

Three KRAS G12D mutant pancreatic adenocarcinoma cell lines purchased from American Type Culture Collection (ATCC, Manassas, VA, USA) were involved in this study: PANC1, SW1990, and ASPC1. ASPC1 (RRID: CVCL_0152) and SW1990 (RRID: CVCL_1723) have homozygous, while PANC1 cells (RRID: CVCL_0480) bear heterozygous KRAS-G12D mutation^82^. All cell lines have been authenticated in the past three years by SNP typing by Multiplexion GmbH.

Cells were cultured in Dulbecco’s Modified Eagle Medium (DMEM; Lonza, Basel, Switzerland) containing 10% fetal bovine serum (EuroClone, Pero, Italy) and 1% penicillin–streptomycin-amphotericin solution (Lonza). The cultures were maintained at 37 ºC in tissue culture flasks in a humidified 5% CO_2_ atmosphere.

All experiments were performed with mycoplasma-free cells.

### Cell proliferation assay

First, doubling time of all the cell lines was defined as previously described^83^. Cell counts were measured after 0, 4, and 7 days using LUNA-II™ Automated Cell Counter (Logos Biosystems, Anyang, South Korea). All measurements were carried out in duplicate for each time point.

Sulforhodamine-B (SRB) assay was carried out to analyze cell proliferation in response to MRTX1133 treatment. Cells were seeded at 7500 cells/well density in a 96-well plate. The following day, the cells were treated with 0, 5, 25, 125, 625, 3125 nM MRTX1133 (Mirati Therapeutics, San Diego, CA, USA; purchased from MedChemExpress, Cat. No.: HY-134813). The cells were treated for two doublings. After the treatment, the wells were washed with Dulbecco’s phosphate-buffered saline (DPBS; Lonza) and fixed with 10% trichloroacetic acid (TCA; Carlo Erba, Milan, Italy) for 1 h at 4 °C. Following washing steps, SRB (Sigma, Saint Louis, MO, USA) dye was applied for 15 min at room temperature. Finally, SRB in excess was rinsed with 1% acetic acid, and protein-bound SRB was dissolved in 10 nM Tris buffer (pH = 7.4). Optical density (OD) was detected at 570 nm using a microplate reader (BioTek 800, Agilent, Santa Clara, CA). Mean background absorbance values were subtracted; data were normalized to control samples and graphed as an average of three independent experiments.

### Clonogenic assay

Clonogenic assay was applied to investigate the long-time effect of MRTX1133 on colony formation. Depending on the cell proliferation rate, 1000–3000 cells were seeded in 6-well plates (Greiner AG, Kremsmünster, Austria) and treated on the next day with 25 nM MRTX1133 for 10 days. Fresh medium with the inhibitor was added on the fifth day. At the end of the experiment, cells were washed with DPBS and fixed with 10% TCA for 1 h at 4 °C. Following washing steps, SRB dye was applied at room temperature for 15 min. After SRB in excess was eliminated by rinsing the plates with 1% acetic acid, protein-bound SRB was dissolved in Tris buffer (10 nM, pH = 7.4). OD was detected at 570 nm using a microplate reader (BioTek 800, Agilent). Data were normalized to controls and shown as an average of three independent experiments.

### Scratch assay

Scratch assay was applied for the investigation of drug effects on cell migration. In 24-well plates (Greiner AG), cells were seeded at 300,000 cells/well density. The next day, cells were treated with 100 nM MRTX1133 for 24 h. Cell monolayers were scratched manually with 10 µl pipette tips. Cellular debris was eliminated by a DPBS washing step and fresh, inhibitor-supplemented medium was added. Wound closure was followed with a zenCellowl incubator microscope (innoME, Espelkamp, Germany) for 24 h, photos were taken every hour. Images were evaluated using ImageJ Fiji software (National Institutes of Health, Bethesda, MD, USA). A modified script originally developed by Suarez-Arnedo et al.^84^ was used to detect the width of the scratches compared to the captured areas of the distinct time points. Data were normalized to the control samples and are shown as a percentage of the t_0_ scratch area. Each cell line was investigated in three biological replicates. T_1/2_ wound closure values were calculated using GraphPad Prism 5 software (Dotmatics, Boston, MA, USA) with non-linear regression (log[inhibitor] vs normalized response, variable slope).

### Boyden chamber assay

PANC1, SW1990, and ASPC1 cells were seeded on 6-well plates (Greiner AG) at 500,000 cells/well density on the first day of the experiments. The next day, the cells were treated with 100 nM MRTX1133 for 24 h. At the end of the treatment, the cells were trypsinized and counted using trypan blue stain to determine the live-dead cell ratio. At the assembly of the Boyden chamber, serum-free DMEM supplemented with 100 μg/ml fibronectin was used as a chemoattractant, and 10 μm thick Nuclepore Track-Etch Membranes (Whatman, Maidstone, United Kingdom) with 8 μm pores were applied for migration filter. Cells were seeded at 30,000 cells/well density in serum-free DMEM with or without 100 nM MRTX1133. Cells were allowed to migrate for 18 h, then the cells were scraped off from the upper side of the membrane. The membrane was then washed with DPBS and fixed in 10% TCA for 1 h at 4 °C. Following fixation, the membrane was repeatedly washed in tap water, then migrated cells were stained with SRB dye for 15 min. Excess dye was removed with 1% acetic acid, then the membrane was photographed, and SRB dye was dissolved in 10 mM Tris buffer to determine OD in a microplate reader (BioTek 800, Agilent) at 570 nm.

### Western blotting

Western blot analyses were applied to investigate protein expression. In 6-well plates (Greiner AG), 300,000 cells/well were seeded and treated with 100 nM MRTX1133 the next day. Following 48 h of treatment, cells were washed with DPBS and fixed with 6% TCA for 1 h at 4 °C. Cells were harvested and centrifuged at 10,000 rcf for 10 min. The supernatant was discarded, and the pellet was dissolved in Läemmli-type buffer including 0.02% bromophenol blue, 10% glycerol, 2% SDS, 100 mM dithiothreitol, 5 mM EDTA, 125 mg/ml urea, 90 mM Tris–HCl, pH 7.9. Qubit 4 fluorometer (Thermo Fisher Scientific, Waltham, MA, USA) was used for protein quantification.

From each sample, 20 µg protein was loaded into 10% polyacrylamide gels. After electrophoretic separation, the samples were transferred to polyvinylidene fluoride membranes. After washing steps using tris-buffered saline supplemented with 1% Tween80 (TTBS), the blocking of nonspecific signs was performed with EveryBlot Blocking Buffer (Bio-Rad Laboratories, Hercules, CA, USA) for 5 min at room temperature. The membranes were incubated in the following primary antibodies overnight at 4 °C: anti-E-cadherin (3195S), anti-N-cadherin (13116S), anti-vimentin (5741S), anti-Snail (3895 T), anti-Slug (9585 T), anti-β-catenin (non-phospho, 19,807), anti-phospho-focal adhesion kinase (p-FAK) (8556), anti-FAK (8557), anti-phospho-extracellular signal-regulated kinase 1/2 (p-Erk) (4376), anti-extracellular signal-regulated kinase 1/2 (Erk) (9102S) (all Cell Signaling Technology, Danvers, MA, USA) for the investigation of epithelial-mesenchymal transition (EMT), along with cell migration, and the mitosis marker anti-phospho-histone H3 (p-H3) to analyze cell proliferation (369-A-14, Cell Marque, Merck KGaA, Darmstadt, Germany). The antibodies were diluted with 5% dry milk or BSA in TTBS according to the manufacturer’s recommendation. The next day, the membranes were incubated in rabbit or mouse secondary antibodies (1:5000, 7074S, and 7076S, Cell Signaling Technology) depending on the source organization of the primary antibody, for 1 h at room temperature. Signal development was carried out using WesternBright ECL HRP Substrate (Advansta Inc., San Jose, CA, USA) and was visualized using an Alliance Q9 device (UVITEC Ltd, Cambridge, United Kingdom). Ponceau total protein staining was performed for normalization.

N-cadherin, E-cadherin, β-catenin, vimentin, Slug and Snail expression have been compared among all the cell lines involved in this study; moreover, a positive control for N-cadherin expression (UO-31 renal cell carcinoma) was also included in this investigation. Quantification was done using ImageLab software (Bio-Rad Laboratories) according to the method of “sum of all data points in a replicate” described by Degasperi et al.^85^. Results are expressed as an average of three independent experiments for each cell line. Brightness and contrast were adjusted in the case of figures displayed in the article, while unmodified blots can be found in Supplementary Materials.

### Experimental animals

NXG female mice were obtained from Janvier Labs (Le Genest-Saint-Isle, France) and were kept in a sterile environment in Makrolon® cages at 22–24 °C (40–50% humidity), with a 12-h light–dark cycle. The animals had free access to tap water and were fed a sterilized standard diet (VRF1, autoclavable, Akronom Kft., Budapest, Hungary) ad libitum. Animals used in our study were taken care of according to the Directive 2010/63/EU of the European Parliament and of the Council on the protection of animals used for scientific purposes. Moreover, the study was carried out and reported in accordance with ARRIVE guidelines. The study has been approved by the ethical committee of the National Institute of Oncology (Budapest, Hungary); furthermore, we confirm that all experiments were performed in accordance with the relevant guidelines and regulations. Animal housing density was according to the regulations and recommendations from the Directive 2010/63/EU of the European Parliament and the Council of the European Union on the protection of animals used for scientific purposes. Permission license for breeding and performing experiments with laboratory animals: PEI/001/1738–3/2015 and PE/EA/1461–7/2020.

### Metastatic mouse models of human pancreatic cancer PANC-1 cell line

Adult NXG female mice, 18 of them, were used in this experiment and kept under the conditions described above. Single-cell suspensions were prepared from PANC-1 monolayer cultures, washed with PBS (BioSera, Boussens, France), and diluted in Medium 199 (Lonza). 80,000 tumor cells were injected in a volume of 10 μl into the spleen of mice, from where metastatic colonies were formed in the liver. Briefly, the splenic injection procedure was as follows: Under anesthesia, the skin was shaved and disinfected with ethanol pads. A 6–8 mm incision was made near the spleen (a left flank incision approximately 2 cm left of the abdominal midline). The spleen was gently exteriorized, and 10 µl of cell suspension was injected into its lower third. The spleen was then carefully reinserted, and the abdominal wall and skin were surgically closed.

Treatments started 7 days after tumor cell injection. MRTX1133 was dissolved in 5% DMSO, 35% PEG300, 15% Tween20, and 45% DPBS in a concentration of 4 mg/ml. Drugs were administered by i.p. injection in a volume of 50 μl per 20 g of mice body weight accounting for 10 mg/kg dose, 5 times per week for 4 weeks. The mice in the control group were treated with the solvent. Two groups with 9 animals per group were established. 14 days after the start of the treatment, two animals in each group were terminated to monitor metastases formation. The final termination of the experiment was 29 days after cell inoculation, i.e., 22 days after treatment started. The mice were euthanized using cervical dislocation in accordance with Directive 2010/63/EU of the European Parliament and of the Council on the protection of animals used for scientific purposes. After the termination of the experiment, the spleens and livers of 7 mice per group were harvested. The antitumor effects of MRTX1133 were assessed by measuring spleen weights and evaluating the tumor-occupied area after vimentin, as well as hematoxylin and eosin staining.

### Histological evaluation of splenic tumors and liver metastases

Formalin-fixed, paraffin-embedded blocks containing the harvested livers and spleens were sectioned. Sections were subjected to hematoxylin and eosin staining. Moreover, tumor cells were stained for anti-human vimentin (1:200, Clone V9, Agilent), anti-human Ki67 (MIB-1 clone, Agilent*),* anti-human E-cadherin (NCl-38, Agilent) and p-Erk (1:200, 4376, Cell Signaling Technology), and chromogen sign was developed by Ultraview-DAB kit performed in an automatic stainer Ventana Benchmark (Ventana, Tucson, AR). The slides were digitized using a 3DHistech Pannoramic® 1000 Digital Slide Scanner (3DHistech Ltd., Budapest, Hungary).

The areas of splenic tumors were measured on the scanned slides using SlideViewer software (3DHistech Ltd.). Mitotic and apoptotic indexes in splenic tumors were defined based on hematoxylin and eosin-stained spleen sections using calibrated 10/10 ocular grid morphometry performed by an experienced pathologist (J.Tímár).

As PANC1 cells showed uniform, strong expression of vimentin, vimentin staining was used for identifying liver metastasis by Qupath software^86^; furthermore, counting metastatic colonies using ImageJ software (National Institutes of Health, Bethesda, MD, USA). Briefly, to investigate the proportion of metastatic areas, scanned images containing the whole liver area were loaded with Qupath and analyzed for cytoplasmic DAB staining using the “Positive cell detection” function. Metastasis formation was also analyzed on hematoxylin–eosin-stained slides with manual training for metastatic cells in Qupath (“Object classification” function). Results were reviewed and approved by a trained pathologist (J.Tímár).

Splenic tumor volume from vimentin-stained sections was defined using the V = (4/3) * π * r^3^ formula, where radius was calculated from the area data (considering splenic tumors as regular circles).

### Histological labeling of ECM

FFPE sections have been labelled for ECM by Mallory-trichrome staining according to the manufacturer’s protocol (Bio-Optica, Milano, It) This stain identifies ECM in a blue colour while cell cytoplasm is red and the nucleus is black.

FFPE sections have been also labelled for collagen fibers by Picrosirius Red staining according to the manufacturer’s protocol (Abcam). This staining identifies collagen fibers in red colour. Histological evaluation of possible changes in ECM composition or morphology were performed by a trained pathologist (J.Tímár).

### Determination of the tumor infiltrating mononuclear cell ratio (TIM)

Intratumoral mononuclear cell definition was created by the International Immunooncology Biomarker Working Group which contains all the intratumoral hematopoietic mononuclear cells excluding leukocytes^87^. The measurement is based on HE slides, which is evaluated by an experienced pathologist (JT) by counting a minimum of 100 cells/area of the primary tumor or metastasis and is expressed in % of TIM in relation to tumor cells. Histological evaluation of changes in the ratio of TIM was performed by a trained pathologist (J.Tímár).

### Statistical analyses

SRB data were analyzed using two-way ANOVA with Bonferroni post-test. In the case of clonogenic assay, unpaired t-test was applied. Results of the immunoblot assays were compared using unpaired t-test with Welch correction. The results of the scratch assay were transformed to a logarithmic scale, and the T_1/2_ wound closure values and statistical significance (*p* ≤ 0.05) were calculated with non-linear regression (log[inhibitor] vs normalized response, variable slope). Statistical significance (*p* ≤ 0.05) was assessed using unpaired t-test or Mann–Whitney test depending on parametric or non-parametric data distribution, respectively. Statistical analyses were carried out by GraphPad Prism 5 software (Dotmatics).

## Supplementary Information

Below is the link to the electronic supplementary material.

Supplementary Material 1

Supplementary Material 2

Supplementary Material 3

## Data Availability

The datasets generated and analyzed in this study are available from the figshare public repository https://figshare.com/s/e59deb89aa5450f55808 (due to the review process, this is a temporary link only, the permanent link will be generated before publication).
