# Supplementary material for: Antimetastatic effects of MRTX1133 KRAS G12D specific inhibitor in a liver metastatic model of pancreatic ductal adenocarcinoma
Source: Sci Rep. 2026 Jan 7;16:4144. doi: 10.1038/s41598-025-34204-y (PMC12859075; doi:10.1038/s41598-025-34204-y)
Supplement: Supplementary file 1 — Supplementary Material 1 [file 41598_2025_34204_MOESM1_ESM.docx]

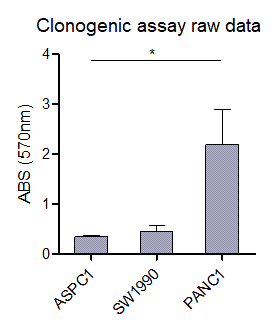


**Supplementary Figure 1. Raw OD data of clonogenic assay.** Control PANC1 cells showed profoundly higher clonogenic potential after 10-day-long MRTX1133 treatment compared to ASPC1 (**p*≤0.05) and SW1990 cell lines.


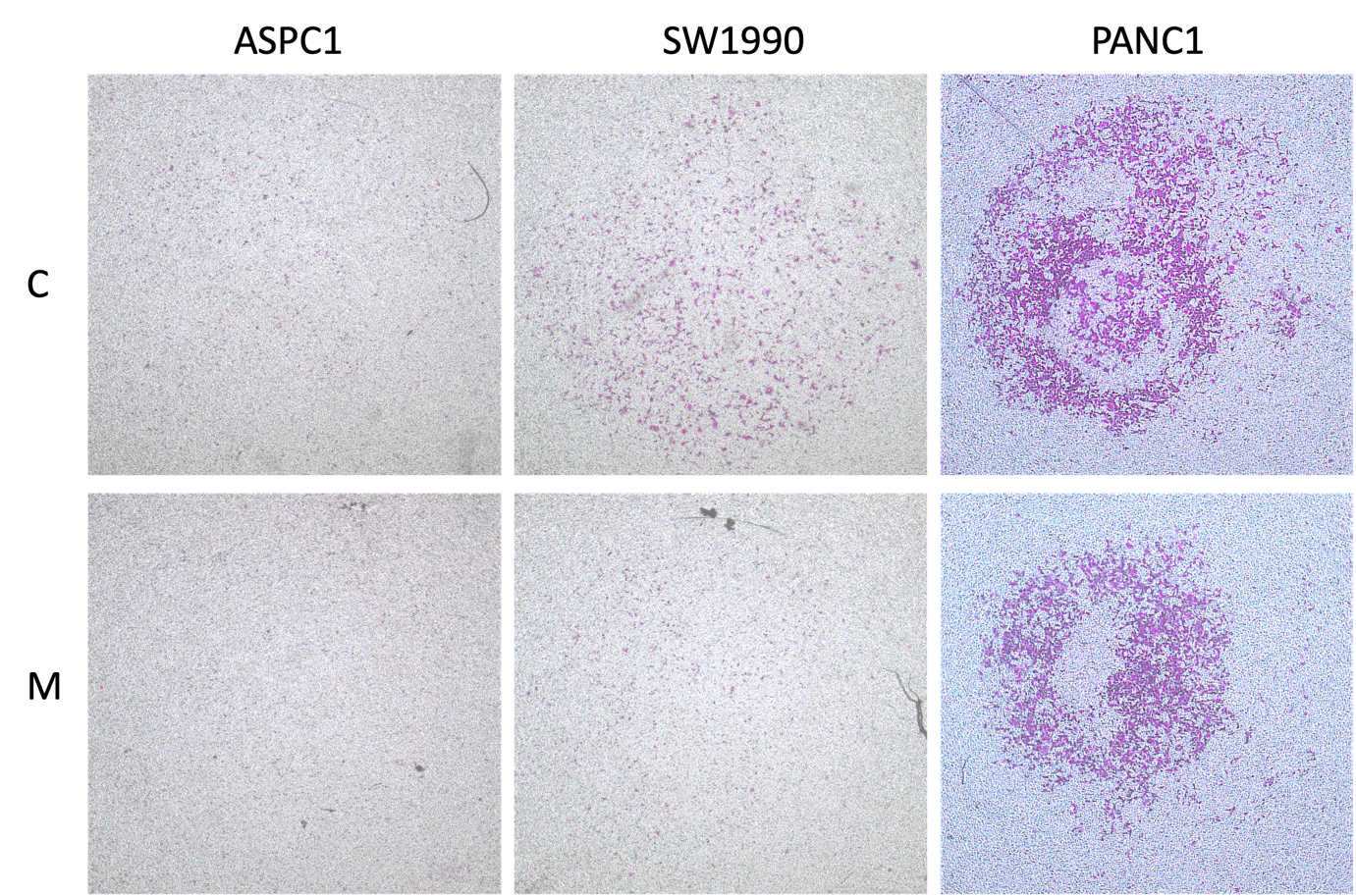


**Supplementary Figure 2. Representative images of Boyden chamber assay performed on control and MRTX1133 treated ASPC1, SW1990, and PANC1 cells.**

C: control samples, M: MRTX1133 treated specimens


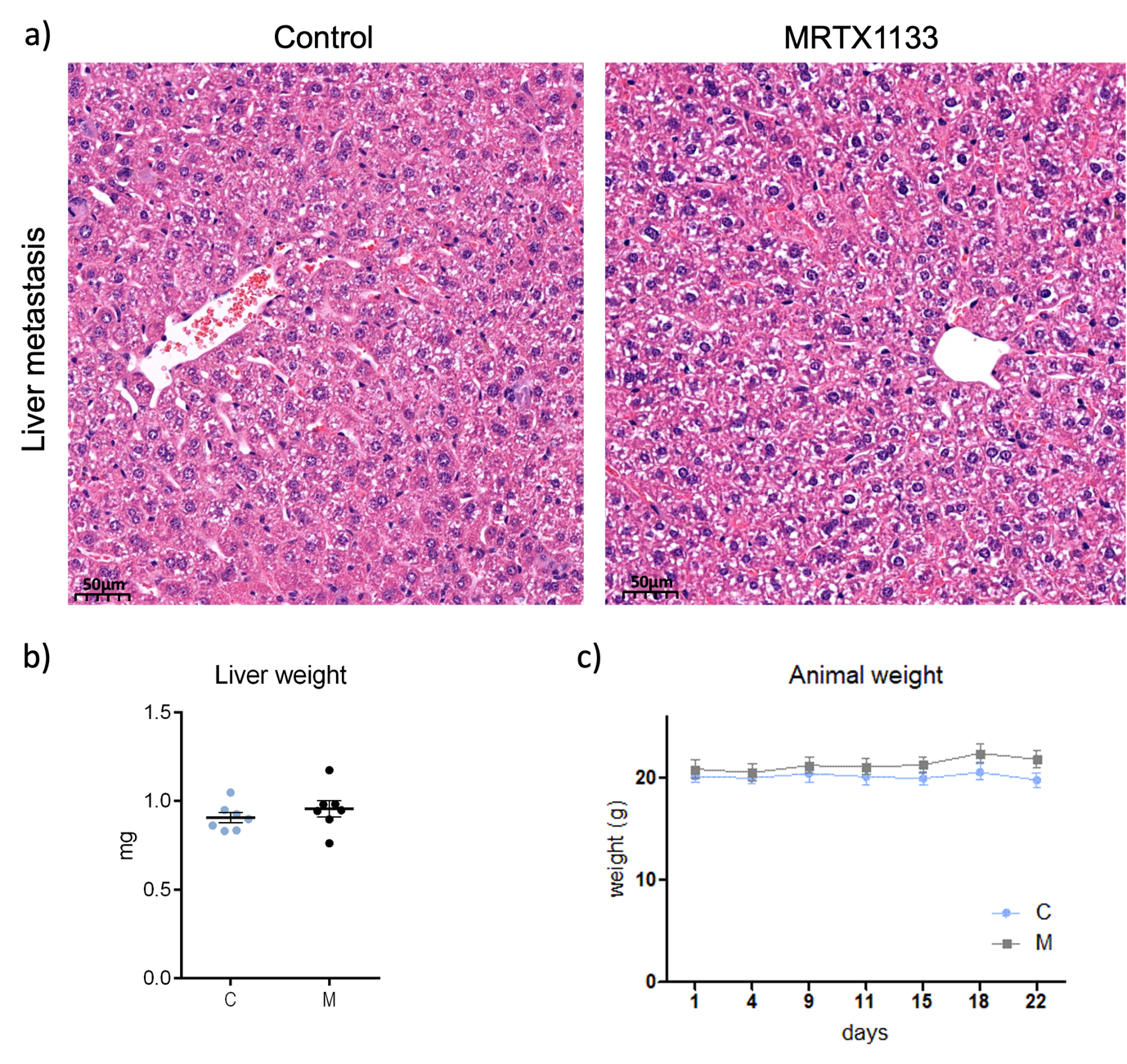


**Supplementary Figure 3. Data associated showing safety features of MRTX1133 treatment**. a) Representative images showing the livers of control (left) and MRTX1133 treated mice. No toxicity related morphological alteration could be observed. b) Liver weights measured following the termination of in vivo experiments. c) The weight of animals was monitored throughout the experiment. No sign of toxicity for 10 mg/kg MRTX1133 dosage could be established based on our data. C: control samples, M: MRTX1133 treated specimens


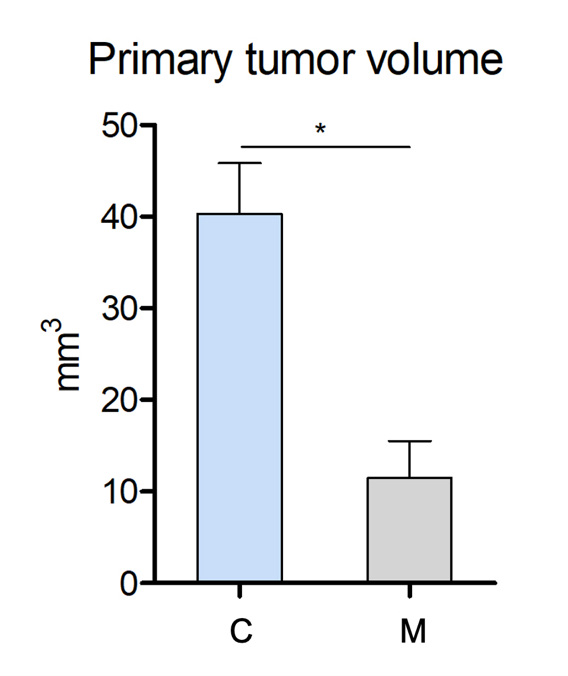


**Supplementary Figure 4. Calculation of the volume of splenic tumors from vimentin-stained sections.** Briefly, the radius was calculated from the area data (considering splenic tumors as circles), then the volume of the tumors was calculated from the radius using V = (4/3) * π * r³ formula.


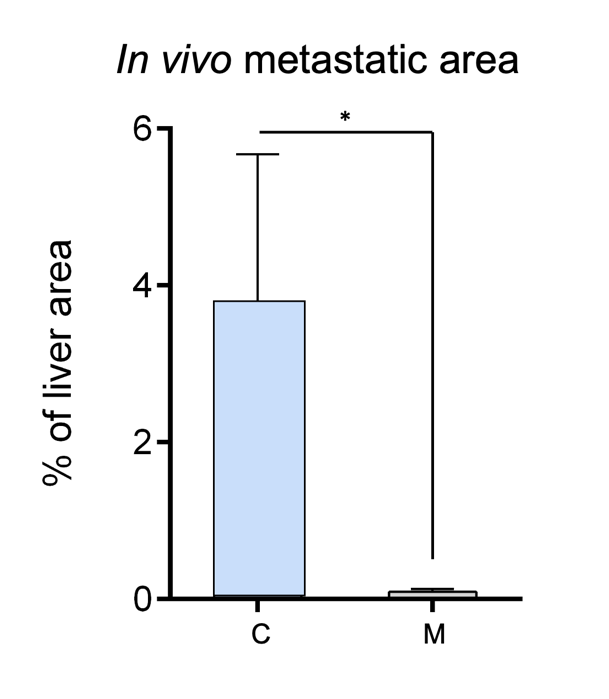


**Supplementary Figure 5. The effect of MRTX1133 treatment on metastatic area based on hematoxylin-eosin-stained slides.** Metastatic area was measured with manual training for metastatic cells in Qupath software using hematoxylin-eosin-stained slides as well. Significantly smaller metastatic regions were detected in the MRTX1133 treated group compared to the control animals (**p*≤0.05). Data represent the mean ± SEM of six replicates. C: control samples, M: MRTX1133 treated specimens


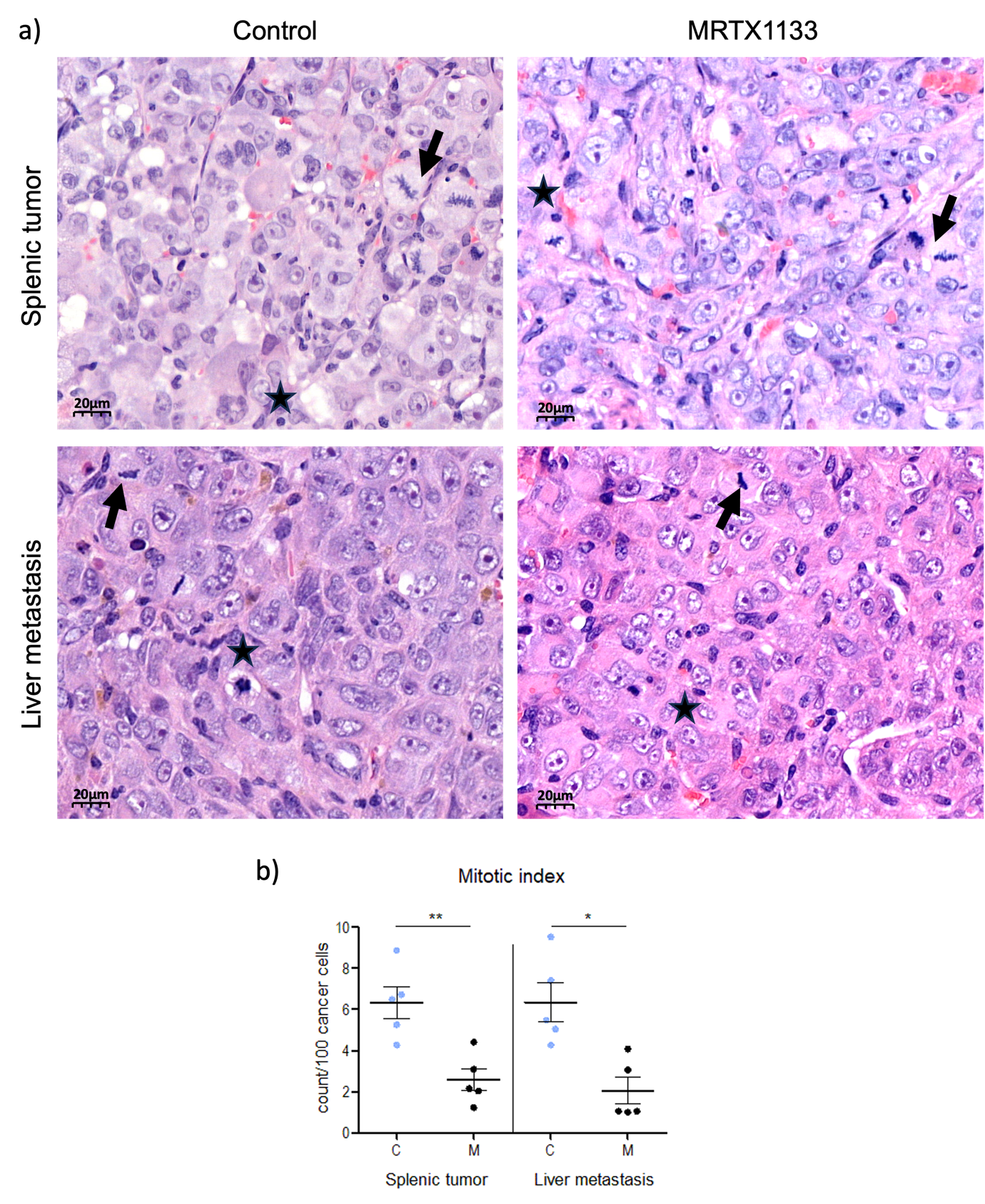


**Supplementary Figure 6. Investigation of mitotic and apoptotic bodies in splenic tumors and metastatic lesions.** MRTX1133 treatment inhibited mitosis both in the splenic and the metastatic tumors compared to untreated tumors. a) Representative images showing mitotic (arrows) and apoptotic bodies (stars) for splenic tumors (pictures in the upper row) and liver metastasis (pictures in the lower row) for control (left column) and mice treated with MRTX1133 (10 mg/kg daily for 22 days, right column). b) Significantly reduced mitotic index were observed in splenic tumors (***p*≤0.01) as well as in liver metastasis (**p*≤0.01) in response to MRTX1133 treatment (B, right). C: control samples, M: MRTX1133 treated specimens


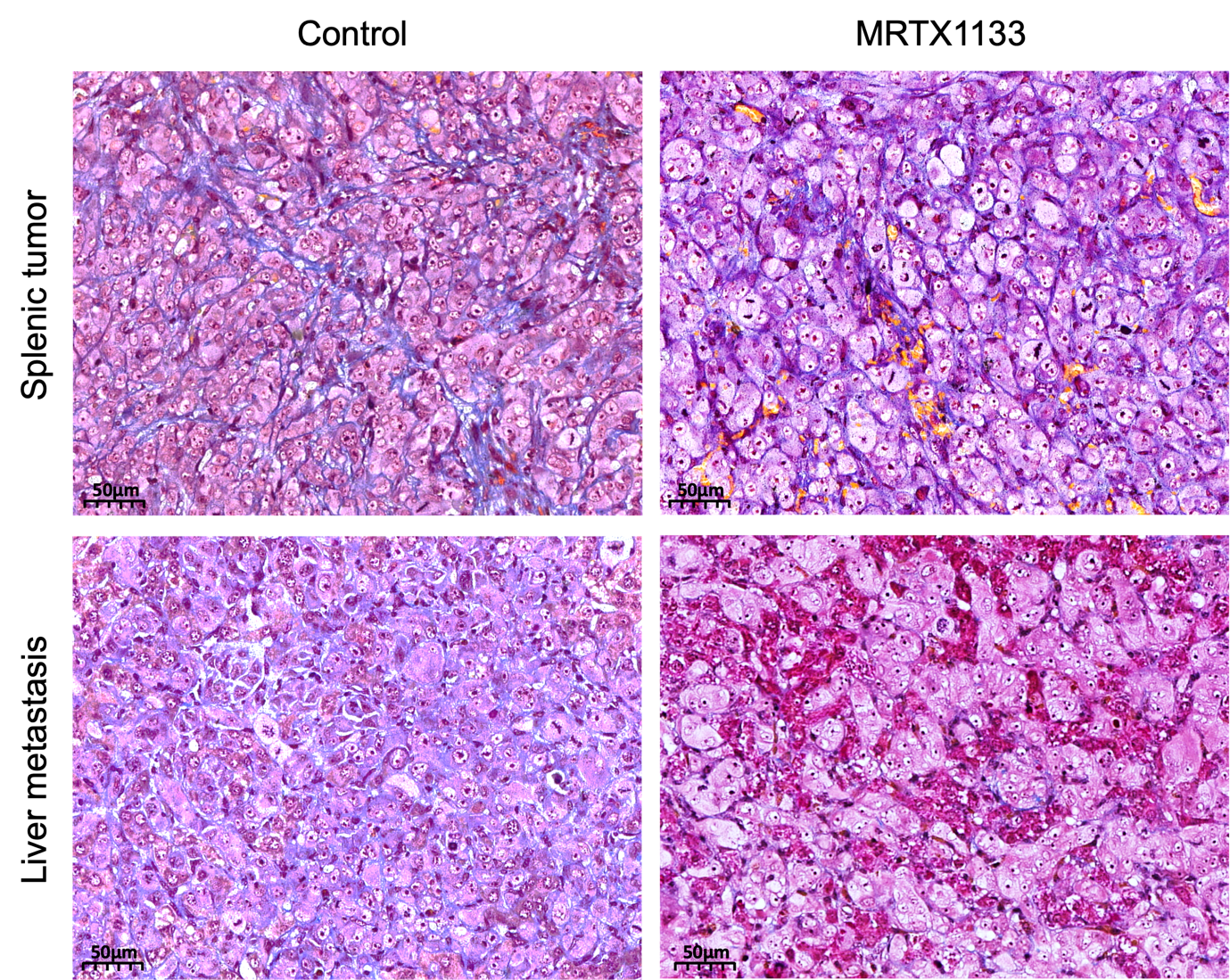


**Supplementary Figure 7. The influence of MRTX1133 treatment on extracellular matrix composition investigated with Mallory’s trichrome** **staining.** Mallory staining detected minimal ECM (collagen stained with blue color) in primary spleen tumors and almost no ECM in liver metastases, with no apparent change upon MRTX1133 treatment.


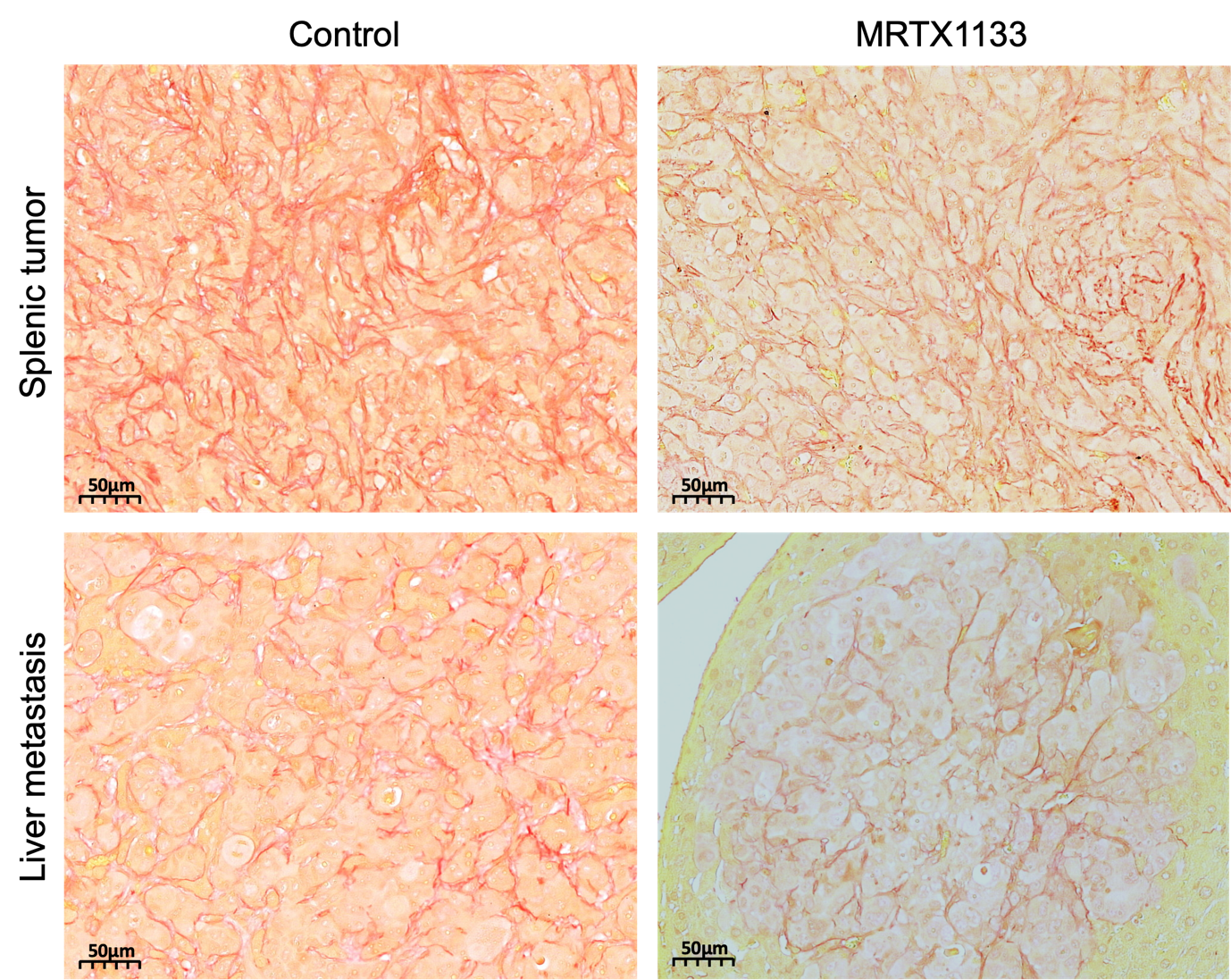


**Supplementary Figure 8. Picrosirius red staining of splenic tumors and liver metastasis.** Picrosirius Red staining is a highly sensitive staining for fibrillar collagen, revealed a collagen-rich matrix in both primary tumors and liver metastases, which can be associated with the activity of cancer-associated fibroblasts. MRTX treatment did not significantly alter collagen content at either location.


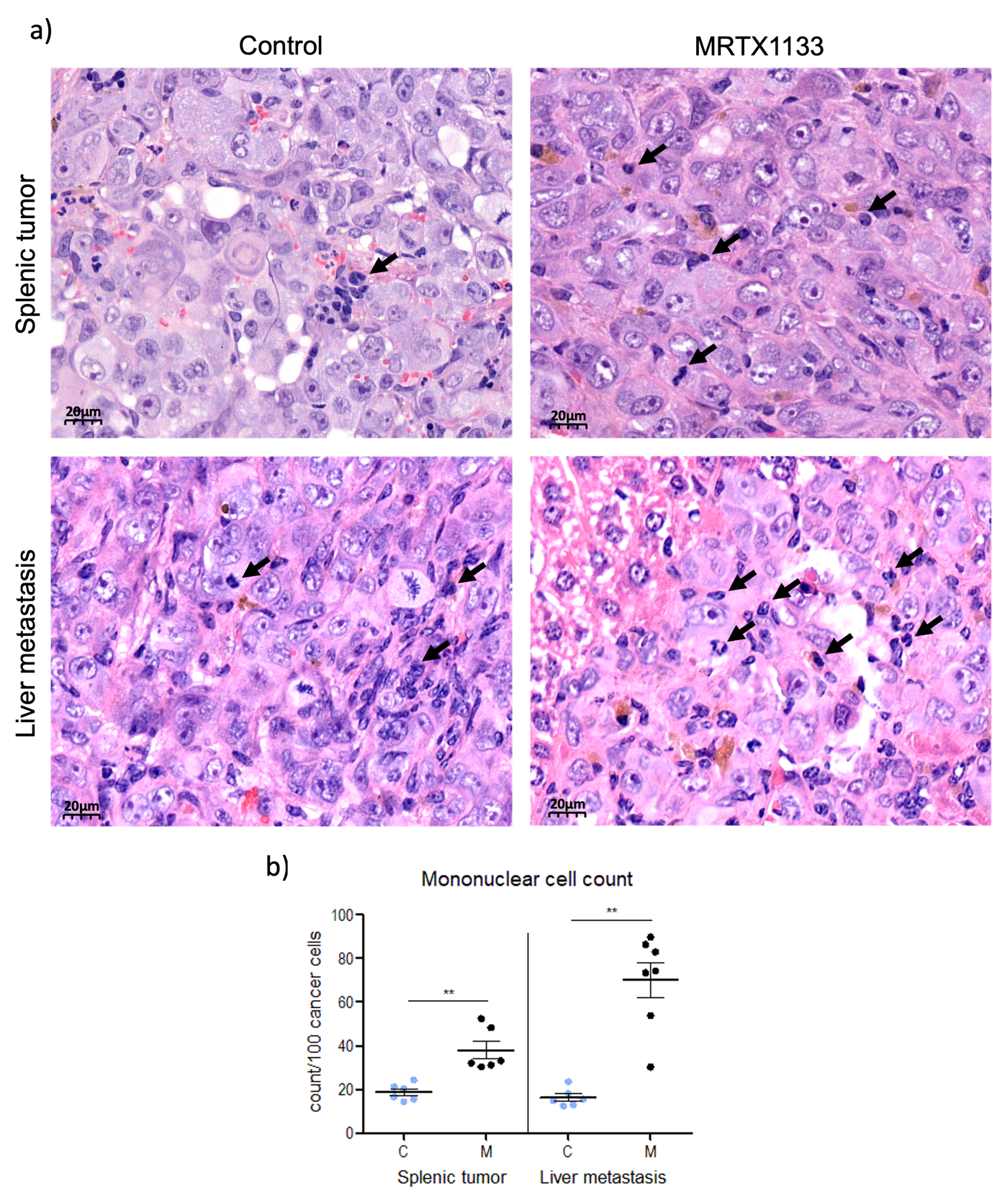


**Supplementary Figure 9. Investigation of mononuclear infliltration of splenic tumors and liver metastases.** a) Representative images of H&E-stained sections showing mononuclear cells in splenic tumors (pictures in the upper row) and liver metastasis (pictures in the lower row) for control (left column) and mice treated with MRTX1133 (10 mg/kg daily for 22 days, right column). b) In control PANC1 spleen primary tumors and in liver metastases the ratio of infiltrating mononuclear cells is relatively low. Interestingly, MRTX1133 treatment led to a significant increase in the TIM ratio in both primary and metastatic lesions (***p*≤0.01), which likely reflects a strong antitumor-effect-associated inflammatory response. C: control samples, M: MRTX1133 treated specimens


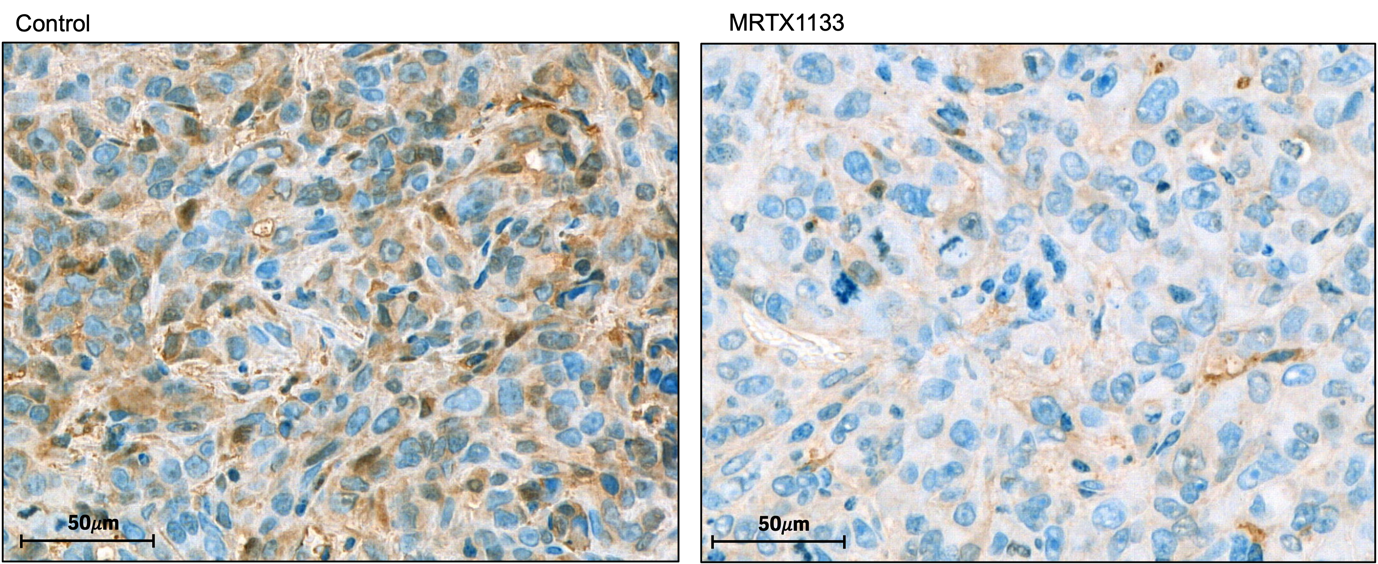


**Supplementary Figure 10. Representative images of phospho-Erk expression in splenic tumor of the control (left) and the MRTX1133 (right) treated mice.** Decreased phospho-Erk expression was observed in splenic tumors of MRTX1133 treated mice compared to untreated ones.


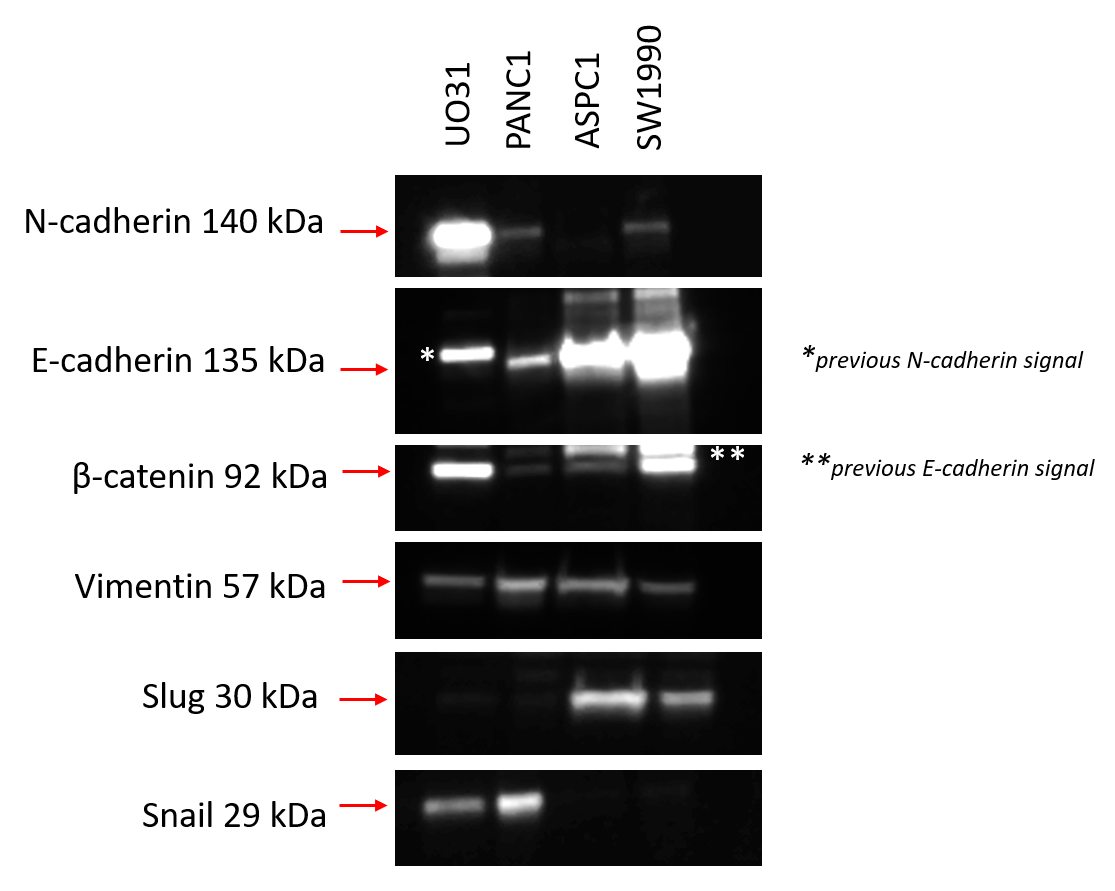


**Supplementary Figure 11. Baseline N-cadherin, E-cadherin, β-catenin, vimentin, slug and snail expression in UO-31, PANC1, ASPC1 and SW1990 cell lines.** Baseline N-cadherin, E-cadherin, β-catenin, vimentin, Slug and Snail expression was investigated in untreated PANC1, ASPC1 and SW1990 cells. UO-31 was included as a positive control for N-cadherin expression.
