# Supplementary material for: Antimetastatic effects of MRTX1133 KRAS G12D specific inhibitor in a liver metastatic model of pancreatic ductal adenocarcinoma
Source: Sci Rep. 2026 Jan 7;16:4144. doi: 10.1038/s41598-025-34204-y (PMC12859075; doi:10.1038/s41598-025-34204-y)
Supplement: Supplementary file 2 — Supplementary Material 2 [file 41598_2025_34204_MOESM2_ESM.docx]

**Supplementary Information**

Supplementary Figures.docx contains the following results:

- Raw OD data of clonogenic assay (Supplementary Figure 1).
- Representative images of Boyden chamber assays (Supplementary Figure 2).
- Data associated showing safety features of MRTX1133 treatment (Supplementary Figure 3).
- Calculation of the volume of splenic tumors from vimentin-stained sections (Supplementary Figure 4).
- The effect of MRTX1133 treatment on metastatic area based on hematoxylin-eosin-stained slides (Supplementary Figure 5).
- Investigation of mitotic and apoptotic bodies in splenic tumors and metastatic lesions (Supplementary Figure 6).
- The influence of MRTX1133 treatment on extracellular matrix composition investigated with Mallory’s trichrome staining (Supplementary Figure 7).
- Picrosirius Red staining of splenic tumors and liver metastasis (Supplementary Figure 8).
- Investigation of mononuclear infliltration of splenic tumors and liver metastases (Supplementary Figure 9).
- Representative images of phospho-Erk expression in splenic tumor of the control and the MRTX1133 treated mice (Supplementary Figure 10).
- Representative images of baseline expression of EMT markers N-cadherin, E-cadherin, β-catenin, vimentin, Slug, Snail (Supplementary Figure 11).

Supplementary material.pptx includes the unmodified blots of immunoblot assays.
